# Spontaneous reoccurrence of *Batrachochytrium dendrobatidis* infections in Australian green tree frogs (*Litoria caerulea*) following apparently successful heat therapy: Case report

**DOI:** 10.1007/s11259-024-10449-2

**Published:** 2024-07-01

**Authors:** Madeleine L. Holmes, Richard Shine, Anthony W. Waddle

**Affiliations:** 1 School of Natural Sciences, Macquarie University, Sydney, NSW Australia; 2 Applied Biosciences, Macquarie University, Sydney, NSW Australia

**Keywords:** *Ranoidea caerulea*, Chytridiomycosis, Elevated temperature, Chytrid detection, qPCR

## Abstract

Heat therapy has been reported as a safe, effective, and readily available treatment method for heat-tolerant frogs infected with *Batrachochytrium dendrobatidis* (*Bd*). We treated wild-caught Australian green tree frogs (*Litoria caerulea*) infected with *Bd* using two periods of elevated ambient room temperature (28.2–30.3 °C for 7 weeks followed by 28.9–34.1 °C for 4 weeks). Frogs exhibited persistent and even increasing infection loads in the first treatment period despite prolonged exposure to elevated temperatures, likely due to the presence of cooler microenvironments within their enclosure (25.5–27.0 °C). All frogs eventually returned negative qPCR tests for *Bd* at the end of the second treatment period, but detectable infections reoccurred one month after frogs were returned to standard housing temperatures (21.2–28.7 °C). Our findings suggest that elevated ambient temperature alone might not eliminate *Bd in vivo* but can reduce infections loads such that they are undetectable by qPCR analysis of skin swabs. Additional factors, such as cooler microenvironments within enclosures or relative humidity, may influence the success of heat therapy. We recommend further research into the combined effects of temperature and humidity during heat therapy and emphasize the importance of accurate temperature measurements as well as post-treatment monitoring at *Bd*-permissive temperatures to confirm successful clearance of infections.

**Supplementary Information:**

The online version contains supplementary material available at 10.1007/s11259-024-10449-2.

## Background

*Batrachochytrium dendrobatidis* (*Bd*) is a fungal pathogen that infects the keratinized skin and mouthparts of amphibians and may cause the disease chytridiomycosis (Berger et al. 1998; Longcore et al. 1999; Voyles et al. 2009). *Bd* has been associated with the extinction of at least 90 species worldwide, including six Australian frog species (Skerratt et al. 2016; Scheele et al. 2017). The impact of chytridiomycosis is typically discussed in the context of wild frog populations, but the disease can wreak havoc on captive populations and in privately owned frogs (Longcore et al. 1999; Alvarado-Rybak et al. 2021).

Antifungal treatments have been developed for *Bd* infections with some treatments showing high efficacy for clearing infections (Woodhams et al. 2012; Baitchman and Pessier 2013). However, antifungal treatment regimens may be both cost and time prohibitive, especially when treating large numbers of animals. Housing heat-tolerant frogs at temperatures at or above 28–30 °C for at least 10 days has been reported as a safe, effective, and readily available treatment method for frog species that can withstand prolonged periods of elevated temperature (Chatfield and Richards-Zawacki 2011; McMahon et al. 2014), but long-term monitoring of frogs after treatments is seldom completed. Because low infection loads can be missed by common swabbing and qPCR techniques (Shin et al. 2014; DiRenzo et al. 2018), negative tests conducted directly after treatments could be false negatives. Long-term monitoring and testing of frogs for the presence of *Bd* following the completion of treatment, at temperatures that favor the pathogen, would provide stronger evidence for the efficacy of prior treatment.

## Case presentation

Between October 2022 and January 2023, 13 adult Australian green tree frogs (*Litoria caerulea)* were collected from a wild population in the Central Coast region of New South Wales, Australia (Online Resource, Table S1). Each frog was initially housed in individual tanks indoors (30 cm W x 20 cm L x 20 cm H) with access to a water dish containing aged tap water at one end and a hide at the other. Frogs were fed six large live crickets twice per week, and water was replaced at least once per week or as required. A skin swab was taken to determine the *Bd* infection status of each frog at the time of capture. Swabbing followed a standardised protocol of 32 strokes with a sterile swab (MW113, Medical Wire and Equipment) per frog; 10 strokes on both the left and right side of the ventral surface, five strokes on each back foot and one stroke on each front foot (Jaeger et al. 2017). Gloves were changed between each frog to prevent sample contamination. We used PrepMan™ Ultra Sample Preparation Reagent to extract DNA from skin swabs (Boyle et al. 2004). Infections were quantified using an optimized quantitative real-time PCR (qPCR) protocol developed by Brannelly et al. (2020). Samples were run in triplicate and were considered positive if *Bd* was detected by amplification of the ITS region in any of the three wells. No *Bd* was detected in any of the samples from the 13 frogs and all were subsequently moved to a large bioactive outdoor enclosure (236 cm L x 120 cm W x 60 cm H) for the purposes of breeding at the end of January 2023.

**Fig. 1:**
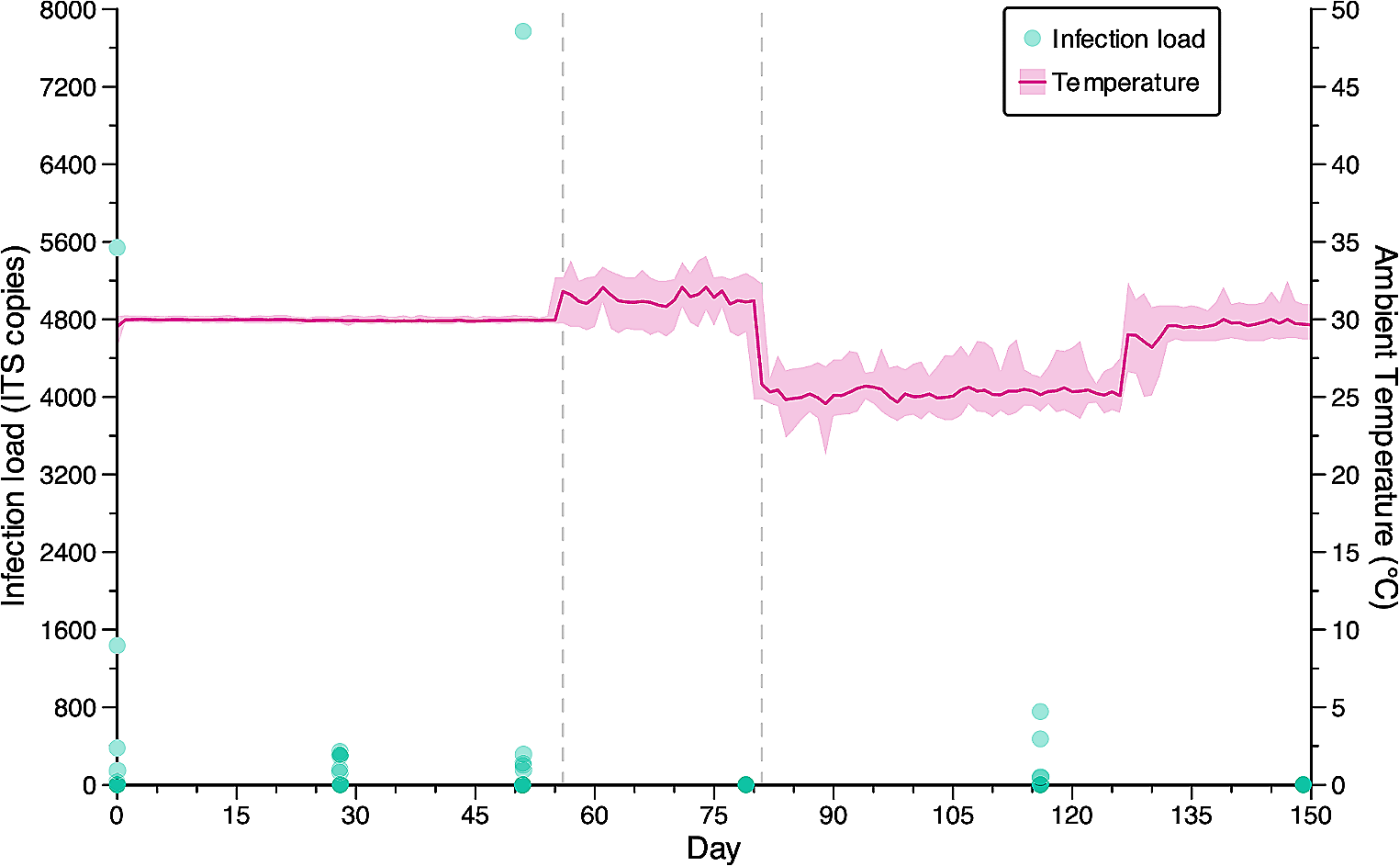
*Bd* infection loads (teal dots, left axis) and daily average ambient room temperature (magenta line, right axis) over time. Teal dots show infection load for each frog (*N* = 13). Shading around the magenta line is the daily temperature range (minimum to maximum). Gray dashed lines indicate days on which the frogs were moved to different rooms

In April 2023, after approximately three months in the outdoor enclosure, three frogs were noted as displaying signs of disease, including abnormal posture and ventral erythema, which are aetiologies consistent with chytridiomycosis (Baitchman and Pessier 2013). Subsequent skin swabs returned positive qPCR results for five of the 13 frogs (mean infection load = 1506 ± 1038 ITS copies, range = 30–5538, Fig. 1). As all frogs had been housed communally, we chose to treat all 13 frogs rather than just those that tested positive, using a heat therapy protocol that had successfully cleared *Bd* infections in green and golden bell frogs (*Litoria aurea*, Waddle 2022; Waddle et al. 2024).

### First heat therapy period

The frogs were housed as a group in a large acrylic enclosure, “Tank A”, (90 cm W x 119 cm L x 60 cm H) containing two clean bricks and three clean terracotta roof tiles in the centre to provide hides, and two clean disposable plastic containers filled with aged tap water at the front of the enclosure that was replaced daily (Online Resource, Figure S1). The enclosure was situated on the floor within a climate-controlled room, set to maintain an average dry-bulb ambient temperature of 30 °C (recorded range = 28.2–30.3 °C). Each frog was visually monitored daily for signs of disease, which had resolved for all frogs by the 14th day. The frogs were swabbed on the 28th day of heat therapy, as we anticipated that it would take no longer than two weeks following the resolution of signs of disease for any subclinical infections to be cleared. Unexpectedly, seven out of 13 frogs tested positive for *Bd* (mean infection load = 267 ± 32 ITS copies, range = 131–343; all frogs appeared healthy, Fig. 1). Treatment was extended for a further three weeks (21 days) before frogs were swabbed and tested again on day 49. The day 49 swabs revealed five frogs with infection loads higher than those of day 28 (mean infection load = 1733 ± 1509 ITS copies, range = 155–7771), including one frog with a substantially higher load than the others (7771 ITS copies, Fig. 1), despite no visible signs of disease (Online Resource, Figure S1).

This increase in infection load prompted us to confirm that all areas of Tank A, and the frogs themselves, were reaching adequate temperatures to prevent *Bd* growth (≥ 27 °C) (Kásler et al. 2022). We placed six Thermochron TC Temperature Logger iButtons that were programmed to log temperature every 15 min into the enclosure. Two of the iButtons were placed within the tiles, two were placed on the floor at the front and the back of the enclosure respectively, and the remaining two were waterproofed using small latex balloons and each placed into a water container. The iButtons remained in place for 24 h. During this time, we used an infrared laser thermometer to record the temperature of each frog every three hours from 1500 to 1800 h on the first day and then from 1000 to 1500 h on the following day. The temperature of the frogs was measured from the center of the dorsal surface (Rowley and Alford 2007).

The average temperature of the two iButtons that were placed on the floor of the enclosure was 27.2 °C (range = 26.5–28.0 °C), whilst the average temperature of the two iButtons placed within the tiles was 26.6 °C (range = 25.5–27.5 °C). The two iButtons placed in the water recorded an average temperature of 26.5 °C (range = 26.0–27.0 °C). Over the same 24 h period the thermostat of the room recorded an average temperature of 30.0 °C (range = 29.31–30.9 °C) (Online Resource, Figure S2). The discrepancy between the temperature recorded by the iButtons and that of the thermostat is likely explained by the height of the thermostat on the wall of the room, as it is situated approximately 1.5 m above the floor where the enclosure was placed. The average temperature of the frogs was 26.3 °C (range = 26.0–27.0 °C), an average of 3.9 °C cooler than the recorded room temperature and approximately 1 °C cooler than the recorded tank temperature (Online Resource, Figure S2), suggesting that the cooler microenvironments within the tiles and water enabled the frogs to regulate their body temperature at slightly below ambient.

### Second heat therapy period

Following the discovery that the enclosure and the frogs were not reaching the desired temperature in the first room, we moved the frogs to a smaller glass enclosure, “Tank B”, (50 cm W x 55 cm L x 60 cm H) within another climate-controlled room with the thermostat set to 32 °C for a further four weeks. The enclosure was placed on a table within the room so that it was directly adjacent to the thermostat to reduce differences between the thermostat temperature and the enclosure temperature. We removed all porous materials (bricks and tiles) from the enclosure as they had created cooler microenvironments within Tank A and are not easily disinfected. Two artificial plastic rock hides were placed in the bottom of the tank. Water containers were replaced with clean containers and clean aged tap water daily from a large water bin that was kept within the room to ensure that the water remained warm. The room maintained a recorded average ambient dry-bulb temperature of 31.4 °C (range = 28.9–34.1 °C) over the 4-week period. After 28 days in Tank B, the frogs were swabbed again, and no *Bd* was detected by qPCR (Fig. 1).

At the time of treatment, we did not collect additional temperature data from within Tank B. However, in May of 2024 we replicated the conditions of this treatment period with five frogs for 24 h to determine the differences between the recorded ambient room temperature and the conditions within the tank. The temperature of each frog was recorded every three hours from 0900 to 1800 h on the second day. The average body temperature of the frogs was 3.4 °C cooler than the recorded ambient room temperature at each spot check. This suggests that during the second treatment period, body temperatures of the frogs may have ranged between ~ 25.5 °C and ~ 28 °C. Further details and summary data from this trial are provided in Online Resource 1.

### Reoccurrence of infection

Tank A was disinfected with F10SC veterinary disinfectant diluted at 1:200, sprayed on all surfaces and allowed to dry completely. Once dry, the enclosure was rinsed with tap water and again left to dry. Enclosure furniture (tiles and bricks) was disinfected with a sprayed 2% bleach solution then washed in 100 °C water for 15 min and left to dry before being returned to the enclosure. The frogs were then moved back into the large enclosure and maintained at standard husbandry temperatures between 21.2 °C and 28.7 °C (Fig. 1). Thirty days after being returned to regular husbandry temperatures, three frogs were noted as displaying non-specific signs of disease (distal ventral hyperaemia and abnormal dorsal skin coloration), and all 13 frogs were swabbed and tested for *Bd* as a precaution. Four of the 13 frogs tested positive for *Bd* (average = 348 ± 166 ITS copies, range = 79–755, Fig. 1). Additional skin swabs were sent to a veterinary pathologist for bacterial culturing and no primary pathogenic bacteria were detected (Online Resource, Figure S3). Ambient temperature of the room was then increased to 28 °C to limit further *Bd* growth and all 13 frogs were moved into individual quarantine tanks (30 cm W x 20 cm L x 20 cm H) with a single plastic hide for anti-fungal treatments with itraconazole. Tank A was again disinfected following the above protocol, with the addition of 2% bleach solution sprayed on all surfaces and left to dry before rinsing with tap water. Enclosure furniture was removed and was replaced with clean plastic hides.

Frogs were kept in isolation and treated following the six-day itraconazole protocol developed by Brannelly (2014). Frogs were given daily five-minute baths in 500mL of 0.005% itraconazole solution in pairs for six consecutive days, before all being returned to the large acrylic enclosure for monitoring. Individual tanks and hides were replaced with a clean tank and hide after each bath during the six-day treatment period. We did not keep the frogs separated after completion of the treatment period as we have observed that they do not tolerate being kept in small tanks for prolonged durations and can injure themselves in their attempts to escape. Skin swabs were then taken 28 days following completion of the treatment protocol, and no *Bd* was detected by qPCR analysis (Fig. 1). Frogs were tested again after 27 weeks and again no *Bd* was detected.

## Discussion and conclusions

Eliminating *Bd* from our captive *L. caerulea* was more difficult than we had expected. We observed persistent and even increasing *Bd* infection loads in the first treatment period at ambient temperatures that have been reported to limit or eliminate *Bd* growth both in vitro and in vivo (Chatfield and Richards-Zawacki 2011; Hettyey et al. 2019; Grogan et al. 2020; Turner et al. 2021). This was likely due to the presence of cooler microenvironments within the enclosure that enabled the frogs to maintain their body temperature lower than the set room temperature, highlighting the importance of ensuring that all areas of an enclosure, as well as the animals themselves, are reaching the necessary temperature rather than relying upon ambient room temperature alone. It is notable that infection loads increased during this period despite frogs being recorded with body temperatures between 26 and 27 °C, which may be due to the specific *Bd* isolate being more tolerant of higher temperatures than is typically reported.

Following the two heat therapy periods, the frogs were housed indoors with biosecurity husbandry protocols in place to prevent the introduction of foreign diseases (Pessier and Mendelson III 2017). It is therefore unlikely that the re-detection of *Bd* represents novel infections. However, approximately one week prior to the reoccurrence of clinical signs many small common odorous ants (*Tapinoma* sp., < 3 mm body length) infiltrated the enclosure and were anecdotally described by animal technicians as being irritating to the frogs. Stress has been associated with increased pathogenicity of *Bd* within various species of frog including *L. caerulea* (Peterson et al. 2013), and this incursion of ants may have been stressful for the frogs, potentially enabling the reoccurrence of detectable *Bd* infections. Alternatively, ants might carry *Bd* zoospores and act as vectors of the pathogen. Toledo et al. (2021) detected *Bd* DNA on *Corethrella* midges caught in the field, and Reinhold et al. (2023) demonstrated that *Culex territans* mosquitoes can carry *Bd* zoospores on their appendages from an infected agar plate to an uninfected agar plate in a controlled lab trial, providing limited evidence that terrestrial invertebrates may be capable of acting as vectors for *Bd*. However, whilst we cannot state with absolute certainty that the ants did not expose the frogs to *Bd*, we do not consider this explanation to be probable. These ants are frequently found infiltrating the enclosure but there have been no further occurrences of *Bd* infections in any of the frogs since the cessation of the itraconazole treatments. Additionally, *Bd* is highly sensitive to desiccation (Johnson et al. 2003) and there is also no evidence to suggest that terrestrial invertebrates can transmit *Bd* across dry substrates.

It is more likely that heat therapy merely reduced infection loads to levels that were undetectable with qPCR, despite the prolonged period of exposure to elevated temperatures. Substantial prior research has demonstrated that lack of detection with qPCR does not necessarily indicate successful clearance of *Bd* (Shin et al. 2014; DiRenzo et al. 2018; Brannelly et al. 2020). The recommended temperature of 28–30 °C for heat therapy in frogs is primarily based on the reported critical thermal maximum (CT_max_) of *Bd* – that is, the temperature at which the pathogen can no longer survive. Whilst numerous in vitro studies have found *Bd* to have a CT_max_ between 28 °C and 30 °C (Piotrowski et al. 2004; Woodhams et al. 2008; Stevenson et al. 2013; Voyles et al. 2017; Grogan et al. 2020; Kásler et al. 2022), results from in vivo studies are less consistent. Only four of thirteen in vivo papers in which elevated temperature was used to treat *Bd* infection in amphibians reported long-term successful clearance of infection in all surviving animals (Table 1). Notably, of these thirteen papers, six did not include a post-treatment monitoring period to confirm that negative test results represented genuine infection clearance, and one had a monitoring period of less than one week (Table 1).

The discrepancy between in vitro and in vivo thermal performance of *Bd* was directly compared by Cohen et al. (2017), who observed high mortality in *Bd-*infected *Atelopus zetecki* housed at 26–28 °C, despite the same *Bd* isolate showing poor performance at 28 °C in vitro. In contrast, Gass et al. (2023) reported high survival in experimentally infected *A. zetecki* housed at 26–27 °C, but only in conditions of high relative humidity (80–90%), with three frogs clearing infection and eight of 10 frogs surviving for over 100 days until the end of the experiment. These opposing survival outcomes within the same species suggest that the role of relative humidity in combination with elevated temperature during heat therapy may have been overlooked, providing one possible explanation for the inconsistency of in vivo outcomes. Relative humidity was not controlled during our treatment of *L. caerulea* and did not reach higher than 77% during the heat therapy period, averaging ~ 50% in both enclosures (Online Resource, Table S2 and Figure S4), and this may have contributed to our unsuccessful clearance of *Bd* infections.

**Table 1:** Summary and outcomes of in vivo publications in which elevated temperature was used to treat *Batrachochytrium dendrobatidis* infections (reverse chronological order)

Species	Infection source	Treatment temp (°C)	Treatment duration	Monitoring period	Reported outcome/s	Reference
*Litoria aurea*	Experimental	32	14 days. Individually treated.	6 weeks	All 22 surviving frogs had no detectable *Bd* infections. One frog died during treatment.	***Waddle et al. *** 2024
*Hymenochirus boettgeri*	Natural & Experimental	20, 30, 25	Water temperature: gradually increased from 20 °C to 30 °C over 10 days, held at 30 °C for 10 days, then gradually decreased from 30 °C to 25 °C over 5 days. Group treated.	Nil	All 20 frogs had no detectable *Bd* infections.	**Carvalho et al. ** 2023
*Atelopus zetecki*	Experimental	26–27 (80–90% relative humidity)	> 100 days. Individually treated.	Nil	3 of 10 frogs had no detectable *Bd* infections. 8 of 10 frogs survived until end of experiment.	Gass et al. 2023
*Rana (Lithobates) chiricahuensis*	Natural	27.5, 29, 32	Water kept at 32 °C and ambient air temp at 29 °C for 6 days, followed by 38 days of 29 °C water and 27.5 °C ambient air temp. Group treated.	218–225 days for 5 individuals	All 38 metamorphs had no detectable *Bd* infections. Five frogs tested after 218–225 days were also negative.^a^	**Heuring et al. ** 2021
*Osteopilus septentrionalis*	Experimental	23, 30	10 days at 23 °C directly after exposure, then 14 days at 30 °C. Individually treated.	Nil^b^	All 13 frogs had no detectable *Bd* infections.	**Sauer et al. ** 2018
*Litoria spenceri*	Experimental	29	4 h per day for 11 weeks. Individually treated.	Nil	10 of 11 frogs had no detectable *Bd* infections by 9th week, 1 frog remained positive at end of experiment.	Greenspan et al. 2017
*Bufo quercicus* and *Osteopilus septentrionalis*	Experimental	23, 30	4 days at 23 °C, followed by 11 days at 30 °C. Individually treated.	Nil	All frogs had no detectable *Bd* infections following each clearance period, for a total of 379 clearances (includes multiple clearances per frog).	**McMahon et al. ** 2014
*Typhlonectes natans*	Natural^c^	32	Water temperature: gradually increased to 32 °C and then maintained for 72 h. Group treated.	6 weeks	All 24 animals had no detectable *Bd* infections following treatment.	***Churgin et al. *** 2013
*Rana (Lithobates) pipiens*	Natural	30, 35	12 h at 30 °C followed by 24 h at 35 °C. Individually treated.	17 days	4 of 4 *Bd* positive frogs had no detectable infections, 1 of 6 negative frogs became infected.	Woodhams et al. 2012
*Rana (Lithobates) catesbieana* and *Acris crepitans*	Natural	30	10 days. Individually treated.	6 days	27 of 28 frogs were *Bd* negative, one *A. crepitans* remained positive.	Chatfield and Richards-Zawacki 2011
*Pseudacris triseriata*	Experimental	32	5 days. Individually treated.	93 days	All 4 surviving frogs cleared infections; 3 frogs died during treatment.	***Retallick and Miera *** 2007
*Mixophyes fasciolatus*	Experimental	27	98 days. Individually treated.	Nil	4 of 8 frogs had no signs of infection at end of experiment.^d^	Berger et al. 2004
*Litoria chloris*	Experimental	37	Two 8 h periods on consecutive days. Individually treated.	> 5 months	All 10 frogs had no signs of infection for more than 5 months following treatment.^d^	***Woodhams et al. *** 2003

‘Infection source’ is whether the infection was naturally acquired in the wild (Natural), or if frogs were infected by researchers (Experimental). ‘Monitoring period’ is time between cessation of heat therapy and subsequent *Bd* testing; ‘Nil’ indicates that frogs were not returned to *Bd*-optimal temperatures following the treatment period, or that there was no reported monitoring period – i.e. frogs were tested for *Bd* clearance immediately following the heat therapy period

Bold references indicate publications that reported successful clearance in all surviving animals, bold and italicized indicates publications that reported successful clearance in all animals with a substantial (> 14 days) post-treatment monitoring period

^a^Animals were collected as tadpoles and metamorphosed during a 60-day quarantine period. Six metamorphosed frogs and one tadpole were sacrificed on the third day for *Bd* testing and two specimens were positive. Heat therapy began on day 16 of the quarantine period. Metamorphs were tested via skin swabs on days 30, 46, and 51

^b^Frogs were placed into thermal gradients ranging between 12 and 33 °C for 2 weeks following heat therapy. Six of the 13 frogs were experimentally re-exposed to *Bd* before being placed into the gradients. As the remaining 7 frogs had access to high temperatures throughout this period, we do not consider this to be a monitoring period. All 13 frogs had no detectable infections after 2 weeks in the thermal gradients

^c^These animals were confiscated by the U.S. Fish and Wildlife Service at a local airport and the source of infection is unknown

^d^Infection status was determined with histopathology techniques rather than qPCR analysis

Although research using heat therapy to clear chytridiomycosis has yielded mixed results, heat therapy is still regularly recommended as an inexpensive, easy treatment option by veterinarians and wildlife groups for heat-tolerant species (e.g. Conservation Evidence (Sutherland et al. 2020), American College of Veterinary Pathologists (ACVP 2023), and Cornell College of Veterinary Medicine (NYS Wildlife Health Program 2018). *L. caerulea* is a commonly kept pet frog species globally, presumably owing to its large size, docile behavior, and attractive coloration. Because frogs obtained from commercial vendors and breeders can harbor *Bd* and subsequently require treatment by pet owners (Waddle et al. 2019), it is essential that veterinarians and wildlife groups be able to provide pet-frog keepers with accurate and up-to-date information regarding safe and effective treatment options for chytridiomycosis. It has been recommended that all wild frogs collected for zoological institutions, captive breeding programs, or research be proactively treated for *Bd* infection prior to being introduced to their regular enclosures and/or housed with other frogs (Young et al. 2007; Forzán et al. 2008). It has also been recommended that wild frogs brought into captivity should not be re-released to the wild without undergoing treatment for *Bd* (Scheele et al. 2014), and that bullfrogs managed within farms be routinely monitored and treated for *Bd* infections (Ribeiro et al. 2019). Facilities that collect and/or house large numbers of amphibians may therefore utilize heat therapy as a treatment method due to the ease and low cost. Additionally, in cases where systematic testing for chytrid is not available, such as at remote field sites, when it is cost prohibitive, or during periods of materials shortages, elevated temperature is more likely to be relied upon for proactive treatments. As heat therapy can falsely appear to have been successful unless frogs are adequately monitored post-treatment, this presents a substantial risk to captive breeding programs if frogs with undetectable infections are prematurely introduced to captive colonies of susceptible and potentially irreplaceable frogs (note: for guidance on effective treatment options see Martel et al. 2018 and Pessier and Mendelson III 2017).

When it is necessary to confirm successful clearance of *Bd*, we recommend that frogs be housed at temperatures that promote optimal *Bd* growth (18–24 °C) for at least 28 days following any treatment protocol, but particularly following the use of elevated temperature only. Negative infection status should be confirmed with two separate swabs taken every 14 days, or, where practical, four separate swabs each taken weekly to account for the poor accuracy of qPCR analysis when infection loads are low, as is to be expected following treatment. In the absence of a post-treatment monitoring period, we caution researchers working with chytrid in amphibians against stating that any treatment has successfully cleared infections, especially when relying only on qPCR analysis of skin swabs for diagnosis. Rather, we suggest that researchers should report that infections were not detected by qPCR analysis of skin swabs following the completion of treatment. Authors should also clearly state the elapsed time between the completion of a treatment and any subsequent skin swabs. Factors such as stress, host skin microbiome, and host-specific immune function should also be reported and discussed when possible.

Additionally, relative humidity should be reported with as much rigor as is currently afforded to temperature (Gass et al. 2023). When relative humidity data are not available, it should be noted if ambient temperature was recorded with a dry or wet-bulb thermometer. The location of data loggers in relation to the animals and their enclosures, and animals’ body temperatures should also be reported. Further investigation into the role of relative humidity in combination with elevated temperature during heat therapy may facilitate the development of more consistently successful heat-therapy protocols.

## Supplementary Information

Below is the link to the electronic supplementary material.

Supplementary file1 (PDF 2084 kb)

## Data Availability

The datasets generated during this case study are available from the corresponding author upon request.
