# Supplementary material for: Spontaneous reoccurrence of Batrachochytrium dendrobatidis infections in Australian green tree frogs (Litoria caerulea) following apparently successful heat therapy: Case report
Source: Vet Res Commun. 2024 Jul 1;48(5):3229–37. doi: 10.1007/s11259-024-10449-2 (PMC11442541; doi:10.1007/s11259-024-10449-2)
Supplement: Supplementary file 1 — Supplementary file1 (PDF 2084 kb) [file 11259_2024_10449_MOESM1_ESM.pdf]

Spontaneous reoccurrence of *Batrachochytrium dendrobatidis* infections in Australian green tree frogs (*Litoria caerulea)* following apparently successful heat therapy: Case report.

Online Resource.

Veterinary Research Communications

Madeleine L. Holmes*[a], Richard Shine[a], Anthony W. Waddle[b]

[a] School of Natural Sciences, Macquarie University, Sydney, NSW, Australia

[b] Applied Biosciences, Macquarie University, Sydney, NSW, Australia

*Corresponding author email: madeleinelholmes@gmail.com

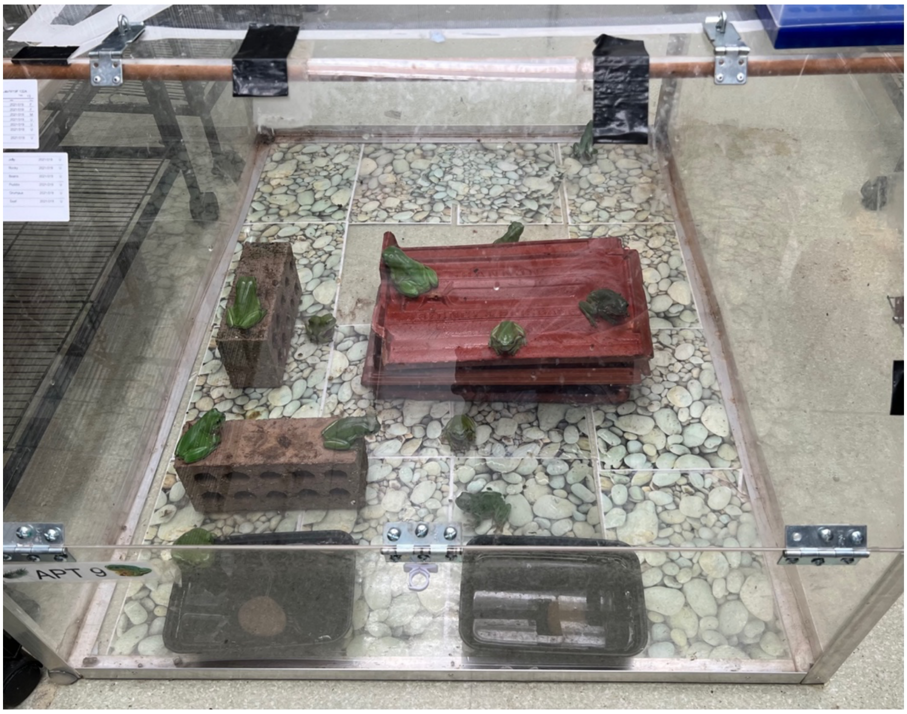

Fig. S1 Photo of the first enclosure set up inside the climate-controlled room on day 49. All frogs appear healthy. Note: only 12 of 13 frogs are visible.

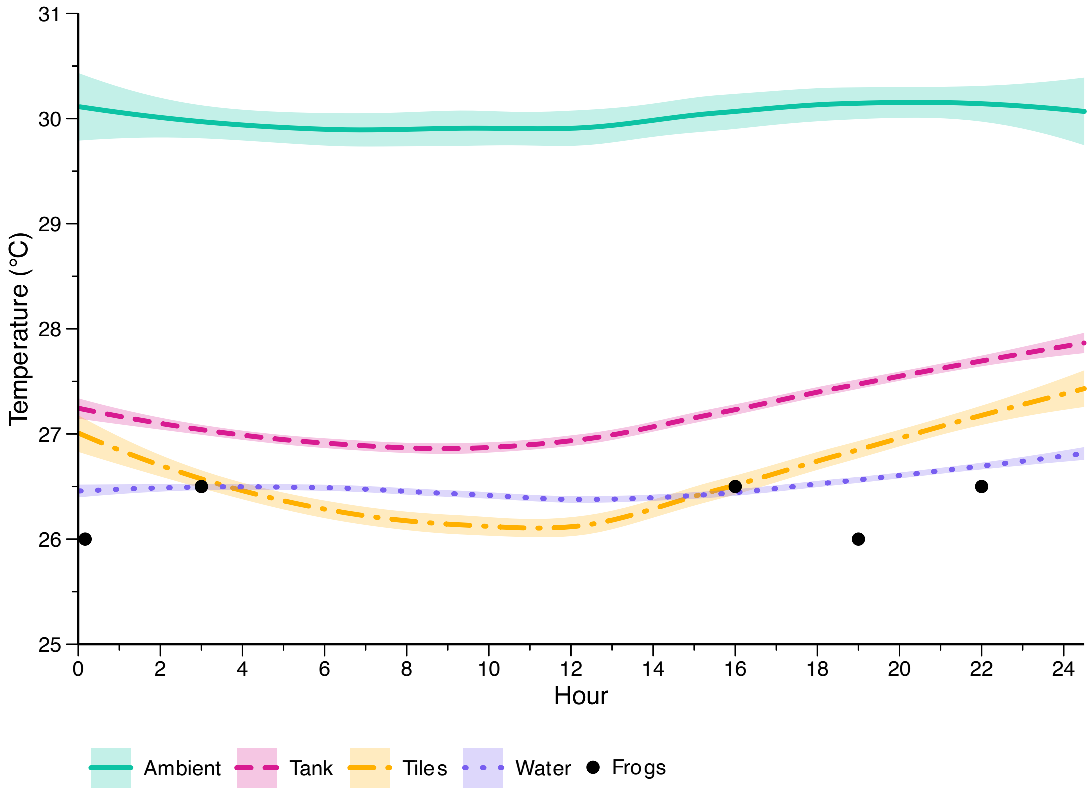

Fig. S2 Average recorded temperatures over a 24-hour period (1500 h - 1500 h) in the first treatment period. Solid teal line is the ambient room temperature as recorded by the thermostat. The dashed pink line is the average temperature in the tank, the dotted blue line is the average temperature in the water, and the dot-dashed yellow line is the average temperature within the tiles, recorded by Thermochron iButtons. Black points show the average temperature recorded by an infrared thermometer pointed at the center of the dorsal surface of the frogs. Shading around the lines shows standard error.

MICROBIOLOGY REPORT - VETERINARY CULTURE

SITE: Skin

SPECIMEN: Swab

GRAM STAIN: Few leucocytes
Scant gram positive cocci

CULTURE Light growth of
Org 1: Staphylococcus species
Org 2: Enterobacter cloacae complex
Org 3: Klebsiella oxytoca

**SUSCEPTIBILITY**	-1-	-2-	-3-		-1-	-2-	-3-
Ampi/Amoxycillin	R	R	R	Doxycycline	S	S	R
Ceftazidime	R	R	S	Sulpha/Trimeth	S	S	S
Gentamicin	S	S	S	Enrofloxacin	S	S	S

No anaerobic bacterial pathogens isolated.

Staphylococcus species further identified as Staphylococcus species kloosii

Final report

Staphylococcus kloosii a coagulase negative Gram positive which is occaisionally isolated from the skin and mucus membranes of domestic and wild animals, specimens from marine animals, and the nasal and oral cavity of birds and reptiles. It is possible that this Staphylococcus can be an opportunistic pathogen in areas exposed to the environment.

Fig. S3 Skin swab bacterial culture microbiology report. No primary pathogenic bacteria were detected. Staphylococcus kloosii was detected and may be an opportunistic pathogen but is not considered to be a primary pathogen.

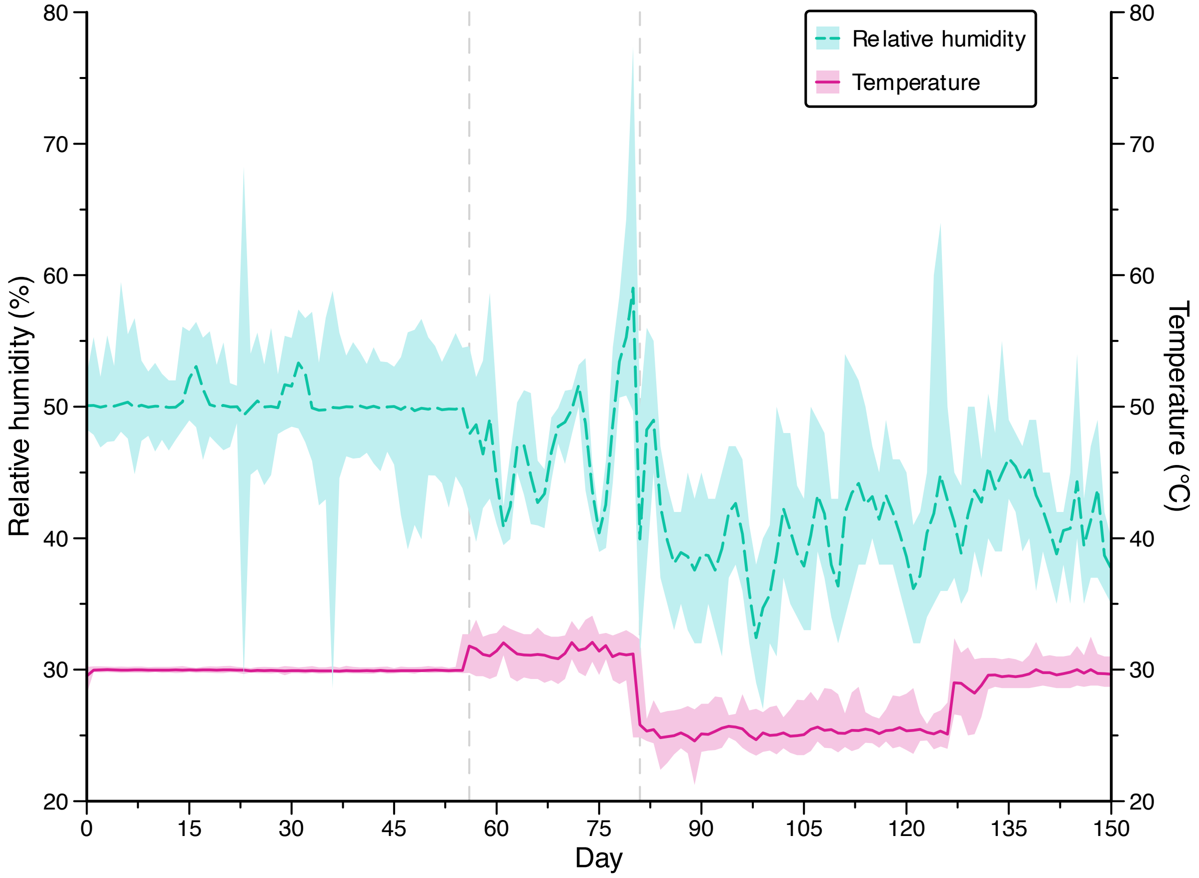

Fig. S4 Daily average relative humidity (dashed line) and ambient temperature (solid line) over time. Shading around the line is the daily range (minimum to maximum). Gray dashed lines indicate days on which the frogs were moved to a different room.

Table. S1 Body mass and snout-urostyle length (SUL) at time of capture for each frog.

Frog number	1	2	3	4	5	6	7	8	9	10	11	12	13
**Body mass (g)**	84.3	65.5	57.3	59.1	56.5	110.0	94.7	55.9	55.0	84.2	58.5	53.1	57.2
**SUL (mm)**	82	76	74	75	78	91	87	76	82	90	82	76	80

Table. S2 Summary of relative humidity (% RH) and ambient temperature (°C) conditions in both rooms throughout the entire duration of heat therapy.

	Room 1		*Room 2*	
	**% RH**	**°C**	**% RH**	**°C**
**Minimum**	28.6	28.2	39.0	28.9
**Maximum**	68.3	30.3	77.4	34.1
**Mean (SE)**	50.3 (0.03)	30.0 (0.00)	47.3 (0.10)	31.4 (0.03)

Additional temperature trial (Tank B)

In May of 2024 we replicated the conditions of the second treatment period with five frogs for 24 hours to determine the differences between the recorded ambient room temperature and the conditions within the tank. We placed six Thermochron iButtons within the enclosure; one in each water container, one in each hide, one at the center of the upper section of the back wall, and one on the center of the lower section of the front wall. The iButtons logged temperature every 15 minutes. Frog body temperature was measured from the center of the dorsal surface with an infrared laser thermometer and every three hours from 0900 – 1800 h on the second day. The average difference between the room thermostat and the walls of the enclosure was 0.9 °C (range = -0.1 – 2.0 °C), the average difference between the thermostat and the hides was 1.8 °C (range = 0.7 – 3.3 °C), and the average difference between the thermostat and the water was 3.7 °C (range = 2.5 – 5.5 °C). We then used these values to calculate the average, minimum, and maximum temperatures that were likely experienced in the enclosure during treatment (Table S2). The average difference between ambient room temperature and frog body temperature at each spot check was 3.4 °C (standard error = 0.5, range = 2.4 – 4.8 °C), similar to that of the water.

Table. S3 Summary of calculated temperatures (°C) for the second treatment period (Tank B)

	**Tank**	**Hides**	**Water**
**Ca. average**	30.5	29.6	27.7
**Ca. minimum**	26.9	25.6	23.4
**Ca. maximum**	34.2	33.4	31.6
